# SyMRI detects delayed myelination in preterm neonates

**DOI:** 10.1007/s00330-019-06325-2

**Published:** 2019-07-08

**Authors:** Victor Schmidbauer, Gudrun Geisl, Mariana Diogo, Michael Weber, Katharina Goeral, Katrin Klebermass-Schrehof, Angelika Berger, Daniela Prayer, Gregor Kasprian

**Affiliations:** 1 Department of Biomedical Imaging and Image-guided Therapy, Medical University of Vienna, Waehringer Guertel 18-20, 1090 Vienna, Austria; 2 Department of Pediatrics and Adolescent Medicine, Medical University of Vienna, Waehringer Guertel 18-20, 1090 Vienna, Austria

**Keywords:** Magnetic resonance imaging, Brain, Newborn, Gestational age, Software

## Abstract

**Objectives:**

The software “SyMRI” generates different MR contrasts and characterizes tissue properties based on a single acquisition of a multi-dynamic multi-echo (MDME)-FLAIR sequence. The aim of this study was to assess the applicability of “SyMRI” in the assessment of myelination in preterm and term-born neonates. Furthermore, “SyMRI” was compared with conventional MRI.

**Methods:**

A total of 30 preterm and term-born neonates were examined at term-equivalent age using a standardized MRI protocol. MDME sequence (acquisition time, 5 min, 24 s)–based post-processing was performed using “SyMRI”. Myelination was assessed by scoring seven brain regions on quantitative T1-/T2-maps, generated by “SyMRI” and on standard T1-/T2-weighted images, acquired separately. Analysis of covariance (ANCOVA) (covariate, gestational age (GA) at MRI (GAMRI)) was used for group comparison.

**Results:**

In 25/30 patients (83.3%) (18 preterm and seven term-born neonates), “SyMRI” acquisitions were successfully performed. “SyMRI”-based myelination scores were significantly lower in preterm compared with term-born neonates (ANCOVA: T1: *F*(1, 22) = 7.420, *p* = 0.012; T2: *F*(1, 22) = 5.658, *p* = 0.026). “SyMRI”-based myelination scores positively correlated with GAMRI (T1: *r* = 0.662, *n* = 25, *p* ≤ 0.001; T2: *r* = 0.676, *n* = 25, *p* ≤ 0.001). The myelination scores based on standard MRI did not correlate with the GAMRI. No significant differences between preterm and term-born neonates were detectable.

**Conclusions:**

“SyMRI” is a highly promising MR technique for neonatal brain imaging. “SyMRI” is superior to conventional MR sequences in the visual detection of delayed myelination in preterm neonates.

**Key Points:**

• *By providing multiple MR contrasts, “SyMRI” is a time-saving method in neonatal brain imaging.*

• *Differences concerning the myelination in term-born and preterm infants are visually detectable on T1-/T2-weighted maps generated by “SyMRI”.*

• *“SyMRI” allows a faster and more sensitive assessment of myelination compared with standard MR sequences.*

**Electronic supplementary material:**

The online version of this article (10.1007/s00330-019-06325-2) contains supplementary material, which is available to authorized users.

## Introduction

Myelin forms a spiral layer around the nerve fibers in the central nervous system [1]. Myelination is the structural basis for fast transmission of information, and is therefore, integral in developmental and neurobiological processes [1, 2]. The myelin sheath is composed of several layers that encompass different biochemical substances, such as lipids and proteins. Above all, the cholesterol fraction allows the detection of myelin in magnetic resonance imaging (MRI) sequences [3–5]. Brain myelination proceeds step-by-step and essential developmental stages can be recorded by MRI [6–8]. Thus, myelin can be visualized on conventional T1- and T2-contrasts and serves as a non-invasive imaging biomarker for brain maturation. Preterm birth interferes with the process of white matter maturation [9, 10], resulting in a delay of myelination [11]. Neonatal brain MRI is sensitive for the prognostic assessment of subtle cerebral pathologies of preterm neonates [12–14]. However, conventional MRI is a highly time-consuming procedure and currently lacks diagnostic sensitivity for the assessment of myelination.

Quantitative T1- and T2-mapping approaches allow the direct estimation of relaxation parameters of specific tissue types [15–18]. In these tissue-specific maps, disturbing influences of T2-weighted signals on T1-weighted images are eliminated and vice versa, which facilitates neuroradiological assessment [19–23]. As it has been demonstrated in children aged 3 months and older, quantitative maps may allow a highly accurate assessment of brain myelination compared with conventional MR images [24]. However, multi-echo mapping sequences have required acquisition times beyond 10 min, thus limiting their application in a clinical neonatal imaging setting [19, 24, 25].

The MR data post-processing software “SyMRI”, combined with a multi-dynamic multi-echo (MDME)-FLAIR sequence, enables the acquisition of different MR contrasts within a vastly shortened examination time compared with standard MRI sequences [26–28]. Moreover, this technique offers the possibility to exhibit and further define myelination through the automated calculation of myelin fractions [26, 29]. Using the quantitative MDME sequence, specific parameters, such as the T1-relaxation constants, the T2-relaxation constants, as well as the proton density (PD) of the examined tissue, can be quantified [16, 28, 30–35]. The MDME sequence acquires all the required parameters for image post-processing in less than 6 min [29]. Extrinsic scan parameters, such as the repetition time (TR), echo time (TE), and inversion time (TI), are not predefined in this procedure, as these factors can be defined and modulated retrospectively [27, 31]. Once intrinsic tissue parameters are acquired and extrinsic scan parameters are defined, “SyMRI” provides T1-weighted, T2-weighted, PD-weighted, as well as inversion recovery contrasts within less than 1 min [29]. Hence, “SyMRI” is advantageous when multiple MR contrasts are needed, which is common in clinical routine. It has been shown recently that image data generated by “SyMRI” are comparable with conventional T1- and T2-images [27, 30]. Apart from conventional MR contrasts, “SyMRI” allows to generate quantitative MR maps within a few seconds [29]. The aim of this study was to evaluate the feasibility of “SyMRI” in the assessment of myelination. For this purpose, we compared brain myelination in preterm and term-born neonates based on visual assessment on conventional MR images and quantitative maps, generated by “SyMRI”. In addition, visual neuroradiological assessment of myelination based on “SyMRI” and conventional T1- and T2-weighted images was compared in a cohort of preterm and term-born neonates.

## Materials and methods

### Ethical approval

The local Ethics Commission approved the protocol of this study, which was performed in accordance with the Declaration of Helsinki.

### Study cohort

A total of 30 preterm and term-born neonates were examined at the Neuroradiology Department of a tertiary care hospital between June 2017 and June 2018. All newborns imaged in this study were referred for MRI examination by the Neonatology Department, Intensive Care Unit. General indications for neonatal MRI included extreme premature delivery (prior to 28 weeks (w) of gestation), intraventricular hemorrhage, hypoxic-ischemic encephalopathy, and epileptic seizures. Table 1 gives a detailed overview of the clinical data of the subjects included in this study. In the majority of cases, premature infants were examined at approximately term-equivalent age. In contrast, term-born neonates were studied post-natally between 2 days and 48 days post-partum. The gestational age (GA) at MRI (GAMRI) indicates the total of GA and post-partum period up to the date of MRI. In terms of preterm neonates, GAMRI is used synonymously with term-equivalent age in this study. The clinical data of the subjects were retrospectively obtained using the electronic patient management system of the hospital.

**Table 1:** Participant demographics

	Neonates
	*n* = 25
	Term-born
	*n* = 7
Clinical characteristics	
Male/female	1/6
GA (w)^a^	39 + 6, SD = 1 + 2
Post-natal period to MRI (d)^b^	17.3, SD = 18.3
GAMRI (w)^a^	42 + 2, SD = 2 + 2
Clinical diagnosis	
Inconspicuous^c^	*n* = 4
Expired infarction^c^	*n* = 1
Subarachnoid hemorrhage/hypoxia^c^	*n* = 1
Subdural/subarachnoid hemorrhage^c^	*n* = 1
	Preterm
	*n* = 18
Clinical characteristics	
Male/female	9/9
GA (w)^a^	25 + 4, SD = 1 + 6
Post-natal period to MRI (d)^b^	94, SD = 27.3
GAMRI (w)^a^	38 + 1, SD = 2 + 6
Clinical diagnosis	
Inconspicuous^c^	*n* = 10
Microbleeding (cerebellar)^c^	*n* = 4
Intraventricular hemorrhage (grade I)^c,d^	*n* = 1
Intraventricular hemorrhage (grade II)^c,d^	*n* = 1
Intraventricular hemorrhage (grade III/IV)^c,d^	*n* = 2

^a^Data represented as mean (weeks (w)) and standard deviation (SD)

^b^Data represented as mean (days (d)) and standard deviation (SD)

^c^Data represented as total number

^d^According to *Deutsche Gesellschaft fuer Ultraschall in der Medizin* (DEGUM) criteria

*GA* gestational age, *GAMRI* gestational age at MRI, *MRI* magnetic resonance imaging

### Image acquisition, MDME sequence, and post-processing

Infants were fed or slightly sedated (chloral hydrate, 30 mg/kg to 60 mg/kg or chloral hydrate, 30 mg/kg combined with midazolam, 0.1 mg/kg) prior to the MRI examination and bedded in a vacuum mattress to prevent movement artifacts. All neonates were examined in the same Philips Ingenia 1.5-T MR system using a standardized neonatal MRI protocol, which consisted of an axial T1 spin echo (SE) sequence, a T2 turbo spin echo (TSE) sequence (in three orthogonal planes), a diffusion-weighted imaging (DWI) sequence, a susceptibility-weighted imaging (SWI) sequence, and a T1 3D sequence. An MDME sequence (one plane) was also acquired, using two repeated acquisition phases [29, 36]. First phase: one slice was saturated by a slice-selective saturation pulse (flip angle, 120°). Second phase: a train of spin echoes was generated for another slice by a series of slice-selective refocusing pulses (flip angle, 180°) and a slice-selective excitation pulse (flip angle, 90°) [28, 29, 36]. The mismatch between the image slice and the saturated slice allowed to acquire a matrix with variable effects of both relaxation rates [29, 36]. T1- and T2-relaxation parameters were estimated by echo trains characterized by different saturation delays [28, 29, 36]. Based on the estimated T1-relaxation constants, the local B1 field was calculated, which was used to correct effects of flip angle deviations [29]. Longitudinal and transverse relaxation parameters as well as B1 also allow to retrieve the unsaturated magnetization, which is needed to calculate the PD [28]. Based on the acquired parameters, “SyMRI” (Synthetic MR AB, Version 8.0.4) was applied to generate quantitative T1- and T2-maps. The generation of quantitative maps was based on the assignment of voxels characterized by ascertained relaxation parameters to a tissue that showed corresponding relaxation parameters [29]. Hence, voxels were assigned according to their relaxation constants to unmyelinated gray and white matter, myelinated structures, as well as cerebrospinal fluid (CSF). A color-coding according to the T1- and T2-relaxation constants allowed an identification of the different tissues, primarily myelin (Supplementary Figure 1). Technical information about the individual sequences are shown in Table 2.

**Table 2:** Neonatal MRI protocol and technical data

Sequence	Plane	FOV (mm)	Voxel size (mm)	Matrix (slices)	TE (ms)	TR (ms)	AT
2D T1 SE	Transversal	120 × 120 × 90	0.83 × 1.05 × 3	144 × 115 × 30	15	400	3:07
2D T2 TSE	Transversal	120 × 120 × 102	0.94 × 1.06 × 3	128 × 113 × 34	140	3000	1:48
2D T2 TSE	Coronal	110 × 110 × 108	0.94 × 1.06 × 3	116 × 103 × 36	140	3000	1:48
2D T2 TSE	Sagittal	120 × 120 × 108	0.94 × 1.06 × 3	128 × 113 × 36	140	3000	1:48
2D DWI	Transversal	200 × 200 × 92	1.14 × 1.15 × 3	176 × 170 × 28	90	4066	1:34
2D SWI	Transversal	170 × 139 × 90	0.85 × 1 × 2	200 × 138 × 90	12	51	3:35
3D T1	Sagittal	120 × 120 × 99	0.75 × 0.75 × 2	160 × 160 × 99	7.6	25	3:46
2D MDME	Transversal	200 × 165 × 109	0.9 × 1 × 4	224 × 159 × 22	13	3309	5:24

*AT* acquisition time, *DWI* diffusion-weighted imaging, *FOV* field of view, *MDME* multi-dynamic multi-echo-FLAIR sequence, *SE* spin echo, *SWI* susceptibility-weighted imaging, *TE* echo time, *TR* repetition time, *TSE* turbo spin echo

### Myelin score and myelin assessment

To determine myelination, we developed a myelin total score (MTS), based on existing MR myelination scores [6, 8, 37]. A total of seven brain regions were evaluated for myelination on axial T1- and T2-imaging data: medulla oblongata, mesencephalon, thalamus, internal capsule, optic tract, frontal lobe (cortical and subcortical white matter), and central region (cortical and subcortical white matter). Based on existing myelination scores, a minimum of zero and a maximum of four points were allocated per region [6, 8, 37, 38]. Criteria about how the points for myelin assessment were allocated are shown in Table 3 and supplementary Figure 1. Regions of interest placement are shown in supplementary Figure 2, based on conventional T1-weighted images. Myelin assessment was performed in the right hemisphere by default. In case of a right-sided pathology, myelin evaluation was performed in the left hemisphere. Subsequently, the values of the individual brain areas were totaled, resulting in MTS values for T1- and T2-imaging data. The myelin assessment was performed by two experienced and independent neuroradiologists (rater 1, 15 years of experience with neonatal MRI and rater 2, 6 years of experience with neonatal MRI), who were blinded to the GAMRI and GA of the neonates. Myelination was evaluated on both conventional T1-weighted images (T1 SE/T1 3D sequence)/T2-weighted images, as well as on quantitative T1-/T2-maps, generated by “SyMRI”. During myelin assessment, rater 1 performed a critical visual review of the image data. Neonates were excluded from the study if myelin assessment was not possible, for instance due to motion artifacts.

**Table 3:** Criteria for the assessment of myelination

Myelination score per region	T1-image^a^	T2-image^b^	T1-map^c^	T2-map^d^
0	Slightly hypointense/isointense to surrounding tissue	Isointense to surrounding tissue	Color-coding corresponding to a T1-relaxation constant^e^ of 1750 to 2000 ms	Color-coding corresponding to a T2-relaxation constant^f^ of 175 to 200 ms
1	Slightly hyperintense to surrounding tissue/hyperintense to CSF	Slightly hypointense to surrounding tissue/hypointense to CSF	Color-coding corresponding to a T1-relaxation constant^e^ of 1500 to 1750 ms	Color-coding corresponding to a T2-relaxation constant^f^ of 150 to 175 ms
2	Hyperintense to surrounding tissue/clearly hyperintense to CSF	Hypointense to surrounding tissue/clearly hypointense to CSF	Color-coding corresponding to a T1-relaxation constant^e^ of 1250 to 1500 ms	Color-coding corresponding to a T2-relaxation constant^f^ of 125 to 150 ms
3	Clearly hyperintense to surrounding tissue/considerably hyperintense to CSF	Clearly hypointense to surrounding tissue/considerably hypointense to CSF	Color-coding corresponding to a T1-relaxation constant^e^ of 1000 to 1250 ms	Color-coding corresponding to a T2-relaxation constant^f^ of 100 to 125 ms
4	Considerably hyperintense to surrounding tissue	Considerably hypointense to surrounding tissue	Color-coding corresponding to a T1-relaxation constant^e^ of < 1000 ms	Color-coding corresponding to a T2-relaxation constant^f^ of < 100 ms

^a^Conventional T1-image

^b^Conventional T2-image

^c^Quantitative T1-map

^d^Quantitative T2-map

^e^Color-coding of the T1-relaxation constants as represented in supplementary Figure 1

^f^Color-coding of the T2-relaxation constants as represented in supplementary Figure 1

*CSF* cerebrospinal fluid

### Statistical analyses

Preterm and term-born neonates were divided into two groups for comparison. Based on the generally accepted division in preterm and term-born newborns, infants included in this study were allocated according to the GA at the time of birth. Thus, all neonates born < 37 w of gestation were allocated to the preterm neonate group, and subjects born ≥ 37 w were allocated to the term-born neonate group [39]. Statistical analyses were performed using SPSS Statistics for Macintosh, Version 25.0 (IBM Corp, 2017) and XLSTAT 2017, Version 20.5 at a significance level of alpha (*α*) = 5% (*p* < 0.05). Graphs were created using XLSTAT 2017, Version 20.5. In order to detect concordances of the myelin measurements and the calculated MTS values of both raters, an intra-class correlation (ICC) analysis was performed. ICC values of 0.75 or above were considered a strong correlation [40]. In case of high concordance, the results of rater 1 are shown. To assess correlations between the MTS and the GAMRI, a Pearson’s correlation analysis was performed. In order to detect statistical differences between the groups, an analysis of covariance (ANCOVA) (covariate, GAMRI) was performed. Preterm and term-born neonate groups were compared using the corresponding MTS, which was assessed by both raters based on conventional T1-/T2-weighted images, as well as on quantitative T1-/T2-maps, generated by “SyMRI” by both raters. Thus, preterm and term-born neonates were compared eight times in total; rater 1: preterm versus (vs.) term-born neonates based on conventional T1- and T2-images, preterm vs. term-born neonates based on quantitative T1- and T2-maps; rater 2: preterm vs. term-born neonates based on conventional T1- and T2-images, preterm vs. term-born neonates based on quantitative T1- and T2-maps.

## Results

### Feasibility of the application of the MR post-processing software “SyMRI”

**Fig. 1:**
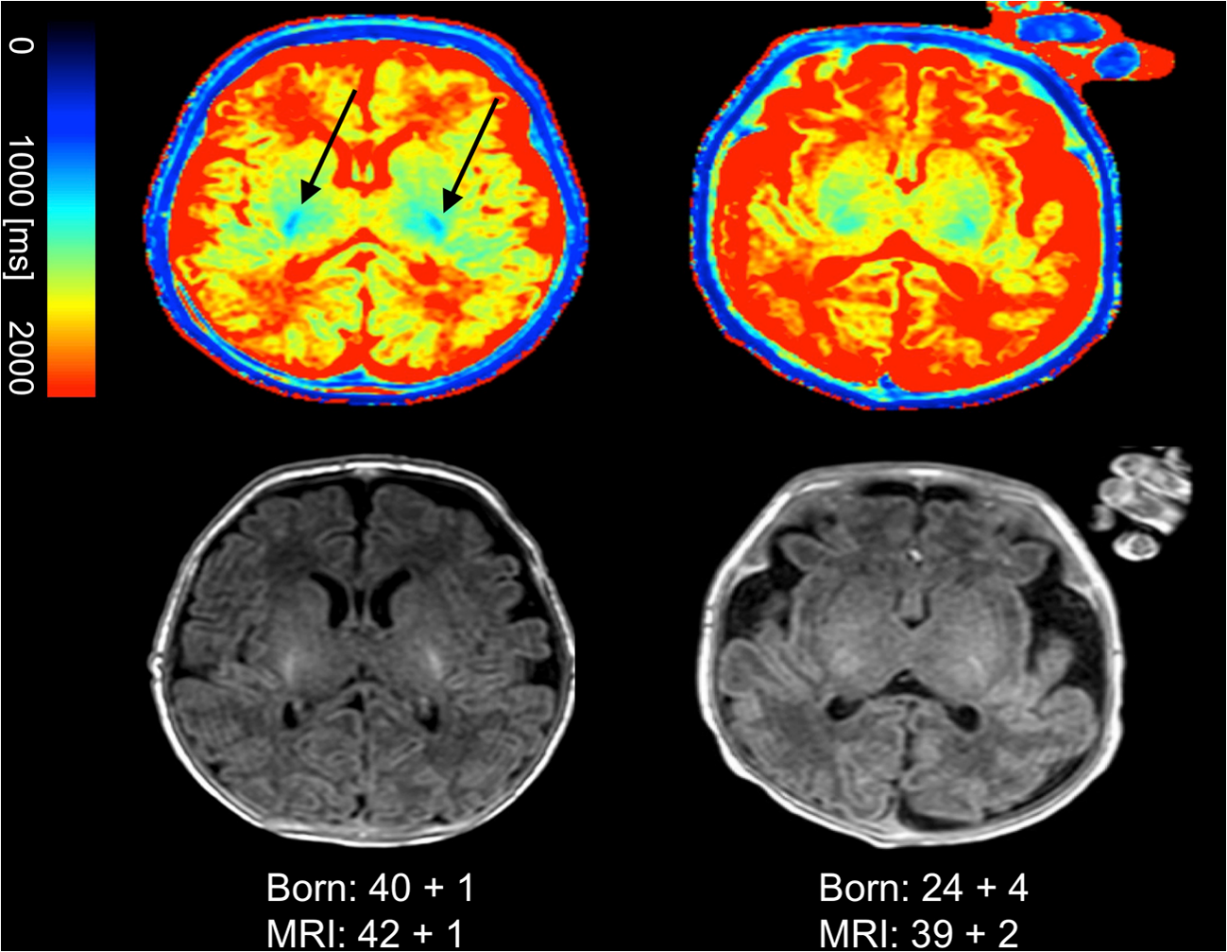
T1-maps, generated by “SyMRI”, are shown in the upper row. Conventional T1-images are shown in the bottom row. The left column shows the quantitative T1-map and the conventional image of a term-born neonate. The right column shows the quantitative T1-map and the conventional T1-image of a former premature infant. T1-relaxation constants are represented by the colored bar. The bluish signal in the posterior limb of the internal capsule in the term-born infant indicates myelin (arrows). The corresponding signal is absent in the premature infant. The color-coding of the tissue in the quantitative maps enables an easier distinction between preterm and term-born neonates compared with conventional images

**Fig. 2:**
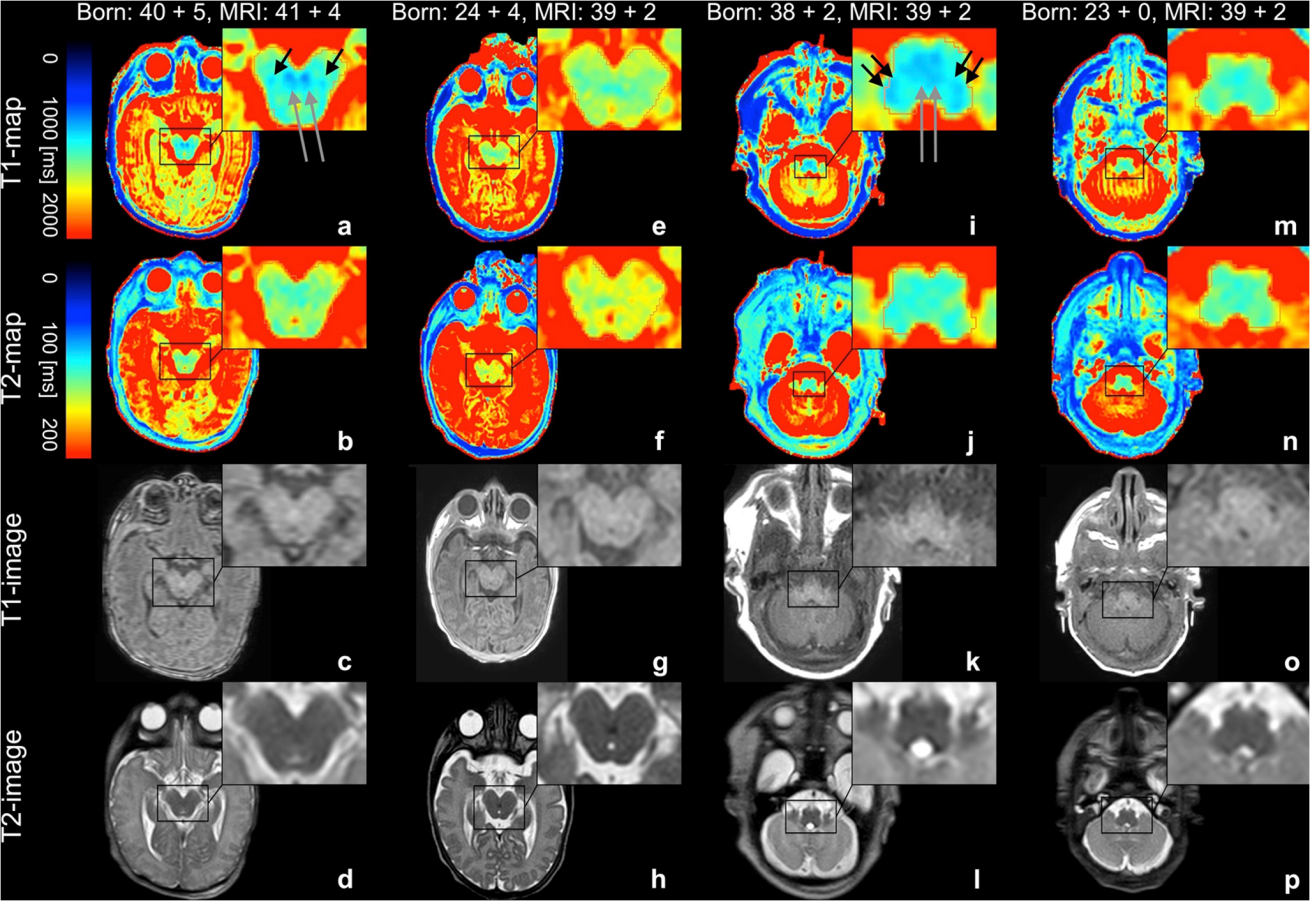
Quantitative T1-/T2-maps are shown in the upper row. T1- and T2-relaxation constants are represented by the colored bars. Conventional T1-/T2-weighted images are shown in the bottom row. **a**–**d** and **i**–**l** Data from a term-born infant. **e**–**h** and **m**–**p** Data from a former premature infant. The quantitative T1-map of the midbrain of the term-born neonate (**a**) shows a distinct myelination of the brachium conjunctivum (long arrows) and medial lemniscus (short arrows). A corresponding signal is far less detectable on the T2-map (**b**) and completely absent in the preterm neonate (**e**, **f**). Conventional MR images of the midbrain are shown for comparison (**c**, **d**, **g**, **h**). The quantitative T1-map of the medulla oblongata of the term-born neonate (**i**) shows a distinct myelination of the inferior cerebellar peduncle (short double arrows) and medial lemniscus (long double arrow). A corresponding signal is far less detectable on the T2-map (**j**) and completely absent in the preterm neonate (**m**, **n**). Conventional MR images of the medulla oblongata are shown for comparison (**k**, **l**, **o**, **p**)

After careful visual review, non-motion-degraded image data were suitable for “SyMRI”-based post-processing in 25/30 subjects (83.3%), 18 preterm (mean GA, 25 + 4 w, SD = 1 + 6 w; mean GAMRI, 38 + 1 w, SD = 2 + 6 w) and seven term-born neonates (mean GA, 39 + 6 w, SD = 1 + 2 w; mean GAMRI, 42 + 2 w, SD = 2 + 2 w). In 5/30 subjects (one preterm and four term-born neonates), myelin assessment using “SyMRI” was not possible; in 1/5 subjects, image quality was too poor for inclusion due to movement artifacts, 3/5 subjects were excluded due to bilateral pathological tissue devastation and 1/5 was excluded due to both poor image quality caused by movement artifacts and a highly devastated brain anatomy. Quantitative T1- and T2-maps of preterm and term-born neonates are shown in Figs. 1 and 2.

### Interrater statistics

There was a high degree of concordance between the T1 and the T2 MTS values assessed by both raters on quantitative MR maps, generated by the MR data post-processing software “SyMRI”: the average measured ICC for the T1 MTS was 0.866 with a 95% confidence interval from 0.696 to 0.941 (*F*(24, 24) = 7.463, *p* ≤ 0.001); the average measured ICC for the T2 MTS was 0.810 with a 95% confidence interval from 0.568 to 0.916 (*F*(24, 24) = 5.251, *p* ≤ 0.001). There was no concordance between the T1 and the T2 MTS values assessed by both raters on conventional MR images: the average measured ICC for the T1 MTS was 0.353 with a 95% confidence interval from − 0.468 to 0.715 (*F*(24, 24) = 1.546, *p* = 0.146); the average measured ICC for the T2 MTS was 0.386 with a 95% confidence interval from − 0.393 to 0.729 (*F*(24, 24) = 1.629, *p* = 0.120).

### Pearson’s correlation analysis

**Fig. 3:**
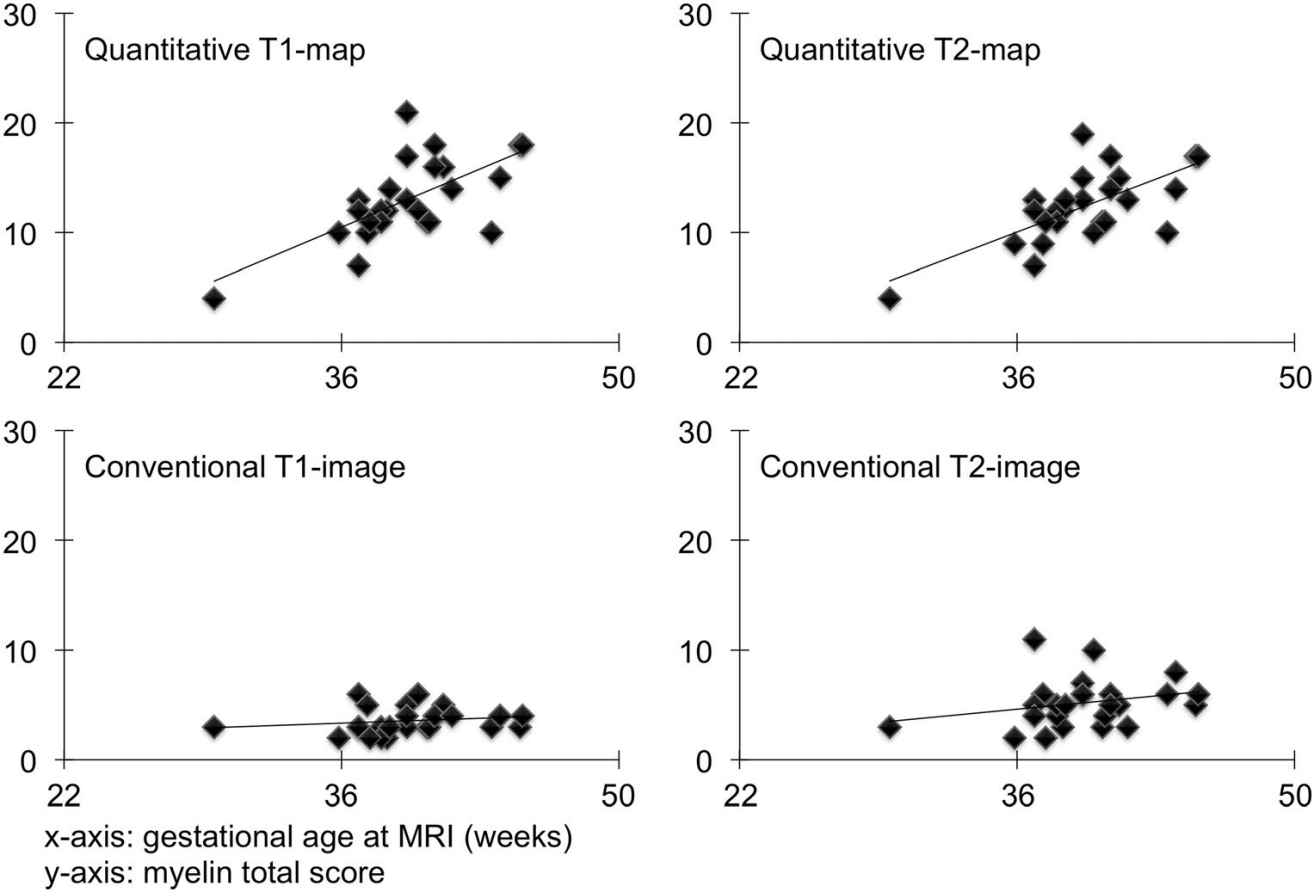
Pearson’s correlation between GAMRI and the T1 (left)/T2 (right) MTS calculated by rater 1. T1-map: *r* = 0.662, *n* = 25, *p* ≤ 0.001; T2-map: *r* = 0.676, *n* = 25, *p* ≤ 0.001. T1-image: *r* = 0.181, *n* = 25, *p* = 0.386; T2-image: *r* = 0.259, *n* = 25, *p* = 0.211

The MTS based on quantitative maps showed a positive correlation (Fig. 3) with the GAMRI (T1: *r* = 0.662, *n* = 25, *p* ≤ 0.001; T2: *r* = 0.676, *n* = 25, *p* ≤ 0.001). The MTS based on standard T1-/T2-weighted images did not correlate (Fig. 3) with the GAMRI (rater 1: T1: *r* = 0.181, *n* = 25, *p* = 0.386; T2: *r* = 0.259, *n* = 25, *p* = 0.211/rater 2: T1: *r* = 0.337, *n* = 25, *p* = 0.100; T2: *r* = 0.199, *n* = 25, *p* = 0.341).

### Preterm vs. term-born neonates

**Fig. 4:**
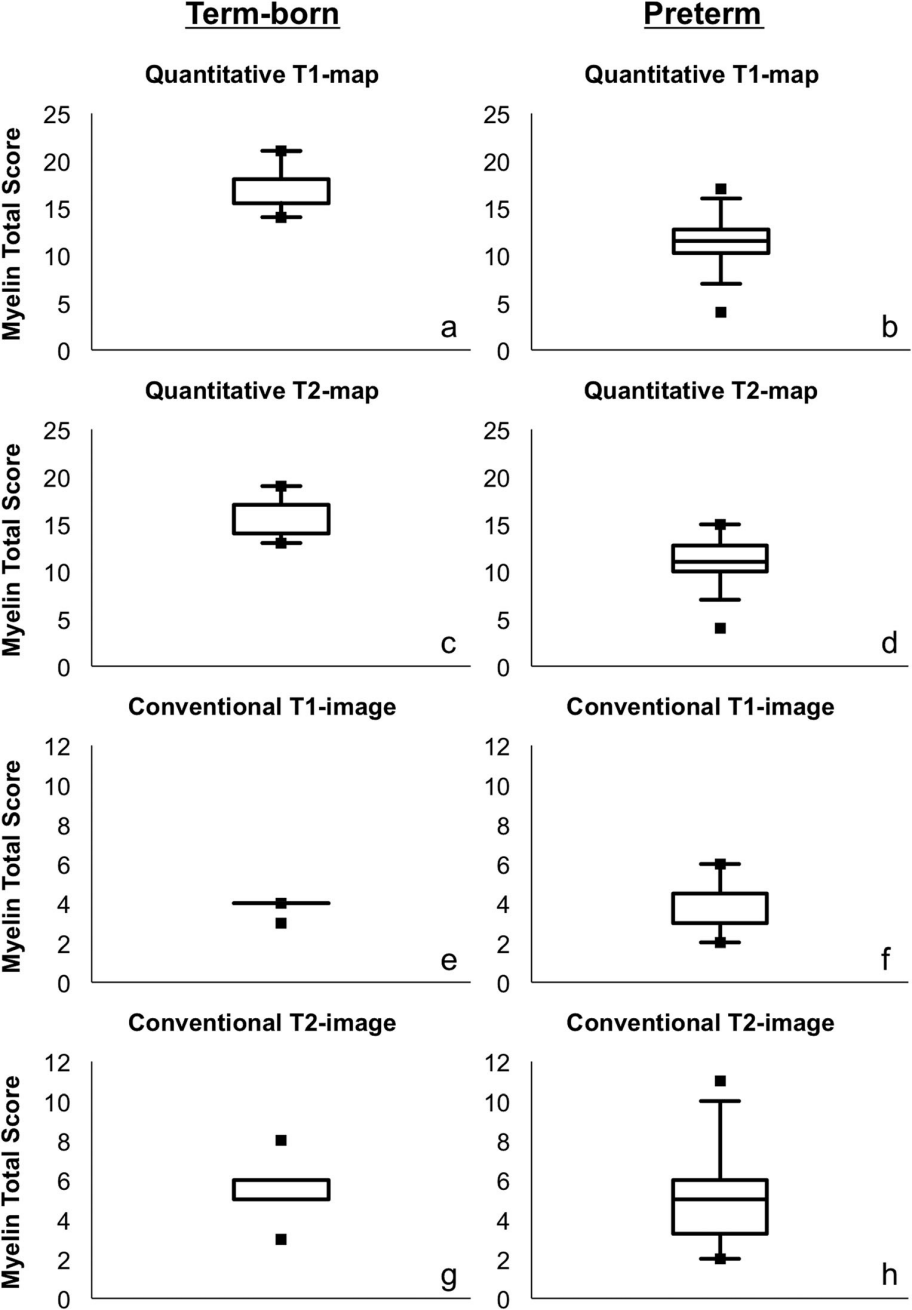
Data from term-born infants are shown in the left column (**a**, **c**, **e**, **g**). The right column shows data from preterm infants (**b**, **d**, **f**, **h**). The boxplots show descriptive data for the MTS values of quantitative T1-/T2-maps (**a**–**d**) and conventional T1-/T2-weighted images (**e**–**h**) assessed by rater 1. Detailed information on the descriptive data is shown in supplementary Table 1

MTS values based on quantitative maps were significantly lower in preterm compared with term-born neonates (T1: *F*(1, 22) = 7.420, *p* = 0.012; T2: *F*(1, 22) = 5.658, *p* = 0.026). No significant differences between preterm and term-born neonates were detectable based on conventional T1-/T2-images (rater 1: T1: *F*(1, 22) = 0.116, *p* = 0.736; T2: *F*(1, 22) = 0.066, *p* = 0.799/rater 2: T1: *F*(1, 22) = 1.141, *p* = 0.297; T2: *F*(1, 22) = 0.045, *p* = 0.834). Descriptive data for the MTS values are shown in Fig. 4 and supplementary Table 1.

## Discussion

Prematurity may be associated with diminished cognitive abilities in up to 35% [41–43] of patients. Myelination positively correlates with higher neurocognitive functions, whereas reduced myelination has been associated with cognitive deficits during later life [44]. In pediatric neuroradiology, the determination of the state of myelination in neonates is currently derived from scores based on the assessment of T1-weighted and T2-weighted MRI sequences [6, 8, 38]. Any tool that ultimately allows for a better characterization of the early stages of myelination may help clinicians to understand and predict cognitive deficits in later life, and thus, identify patients in need of intensified therapeutic support and closer follow-up. In this study, a novel MRI technique—“SyMRI”—was successfully applied in preterm and term-born neonates. The “SyMRI” software creates maps based on T1- and T2-relaxation constants and the PD, which differ depending on the measured tissue [16, 19, 28, 33–35, 45–47]. This facilitates the visualization of different tissue types and enables the quantification of myelin, resulting in a better assessment of brain development and demyelinating diseases [29, 48]. In the present study, “SyMRI” was more efficient and superior to conventional MRI in the visualization of myelin and in the detection of delayed myelination in preterm neonates. Two radiologists independently rated images to consistently detect subtle differences in myelin content in preterm compared with term-born neonates, which were not detectable on conventional T1- and T2-weighted sequences, using “SyMRI”-generated quantitative maps. Myelin could be better distinguished from non-myelinated gray and white matter using quantitative maps than by conventional images (Figs. 1 and 2). The advantages of “SyMRI”-based maps became particularly evident when assessing myelination of brain stem structures, such as the superior and inferior cerebellar peduncles and the medial lemniscus (Fig. 2), since these structures are already myelinated at the normal, expected due date [6, 38]. Numerous studies have examined brain maturity in preterm neonates based on conventional MR images [49–51]. The results of these studies often varied, indicating a lack of reliability in the assessment of neonatal brain maturity and, in a broader sense, unreliable information regarding neurodevelopmental outcome based on standard T1- and T2-weighted images [43, 49]. Various promising imaging techniques have been studied in recent years to assess brain maturity and myelin content, especially diffusion tensor imaging (DTI), DWI, computational morphometry, and resting-state functional MRI [43, 49]. Based on fractional anisotropy values, a significantly reduced myelination of the brain of premature infants was demonstrated on the basis of DTI data [52]. Several studies detected alterations of functional connectivity in preterm compared with term-born neonates, which might be caused by decelerated brain maturation [53–55]. However, the above-mentioned imaging methods are highly time-consuming and have not been studied on an individual or routine patient-level basis [26–28]. Especially in clinical practice, time is an important parameter. Conventional methods for MR mapping would take between 15 and 30 min, even by means of modern techniques [19, 29, 56]. “SyMRI” is a quantitative imaging method that detects myelination-related differences on an individual level, beyond the neonatal brain imaging protocol. Moreover, it generally allows a reduction of examination time while providing multiple MR contrasts, including T1, T2, PD, and inversion recovery. Thus, “SyMRI” offers the possibility to provide quantitative maps as well as various MR contrasts in one-third of the time needed for conventional methods for quantitative mapping. This opens further possibilities in diagnostic neonatal brain imaging, which were not the subject of the present study. Our study has several limitations. “SyMRI”-generated maps and conventional MRI differed in resolution and slice thickness, which limits a direct comparison with a certain extent. The investigated cohort, consisting of 18 preterm and seven term-born neonates, is small and heterogeneous, with different referral reasons. Not all examined infants showed unremarkable brain findings. It must, therefore, be assumed that changes in myelin content were partly related to brain pathologies in both included groups of preterm and term-born neonates. However, this limitation did not influence the clinical radiological assessment or the primary outcome of the study. Although the assessment of myelination using quantitative maps and conventional imaging data is a matter of subjective judgment by the evaluating radiologist, this study was conducted as a qualitative assessment alone, as this most closely resembles clinical practice. This study mainly addressed the added potential of “SyMRI” in the assessment of myelin-associated signal changes. The accuracy and reliability of “SyMRI”-generated T1-weighted and T2-weighted images in the detection and analysis of neonatal brain injuries (hypoxic-ischemic injury, infection, trauma, etc.) was outside the scope of this study, but should be the topic of future investigations.

In summary, our results indicate that assessing the maturity and myelination of the neonatal brain using quantitative MR maps is a highly viable, reliable, and easy-to-apply method. Compared with conventional T1- and T2-weighted images, the myelin content can be assessed more accurately, within a highly reduced examination time. We conclude that quantitative T1- and T2-maps allow a rapid and valid assessment of brain myelination, and therefore provide a highly reliable imaging biomarker.

## Electronic supplementary material

ESM 1 (DOCX 796 kb)

## Abbreviations

CSF: Cerebrospinal fluid
DTI: Diffusion tensor imaging
DWI: Diffusion-weighted imaging
GA: Gestational age
GAMRI: Gestational age at MRI
ICC: Intra-class correlation
MDME: Multi-dynamic multi-echo-FLAIR sequence
MRI: Magnetic resonance imaging
MTS: Myelin total score
PD: Proton density
SE: Spin echo
SWI: Susceptibility-weighted imaging
TE: Echo time
TI: Inversion time
TR: Repetition time
TSE: Turbo spin echo
