# Supplementary material for: SyMRI detects delayed myelination in preterm neonates
Source: Eur Radiol. 2019 Jul 8;29(12):7063–72. doi: 10.1007/s00330-019-06325-2 (PMC6828642; doi:10.1007/s00330-019-06325-2)
Supplement: Supplementary file 1 — (DOCX 796 kb) [file 330_2019_6325_MOESM1_ESM.docx]

**Supplementary data**

**Supplementary Table 1:** Descriptive data for the MTS values assessed by rater 1

| ***n* = 25:** | **Term-born: *n* = 7** | | | | **Preterm: *n* = 18** | | | |
| --- | --- | --- | --- | --- | --- | --- | --- | --- |
| **Modality:** | T1-map^a^ | T1-image^b^ | T2-map^c^ | T2-image^d^ | T1-map^a^ | T1-image^b^ | T2-map^c^ | T2-image^d^ |
| Minimum | 14 | 3 | 13 | 3 | 4 | 2 | 4 | 2 |
| Maximum | 21 | 4 | 19 | 8 | 17 | 6 | 15 | 11 |
| 1^st^ quartile | 15.5 | 4 | 14 | 5 | 10.25 | 3 | 10 | 3.25 |
| Median | 18 | 4 | 17 | 6 | 11.5 | 3 | 11 | 5 |
| 3^rd^ quartile | 18 | 4 | 17 | 6 | 12.75 | 4.5 | 12.75 | 6 |

^a^ Quantitative T1-map

^b^ Conventional T1-image

^c^ Quantitative T2-map

^d^ Conventional T2-image


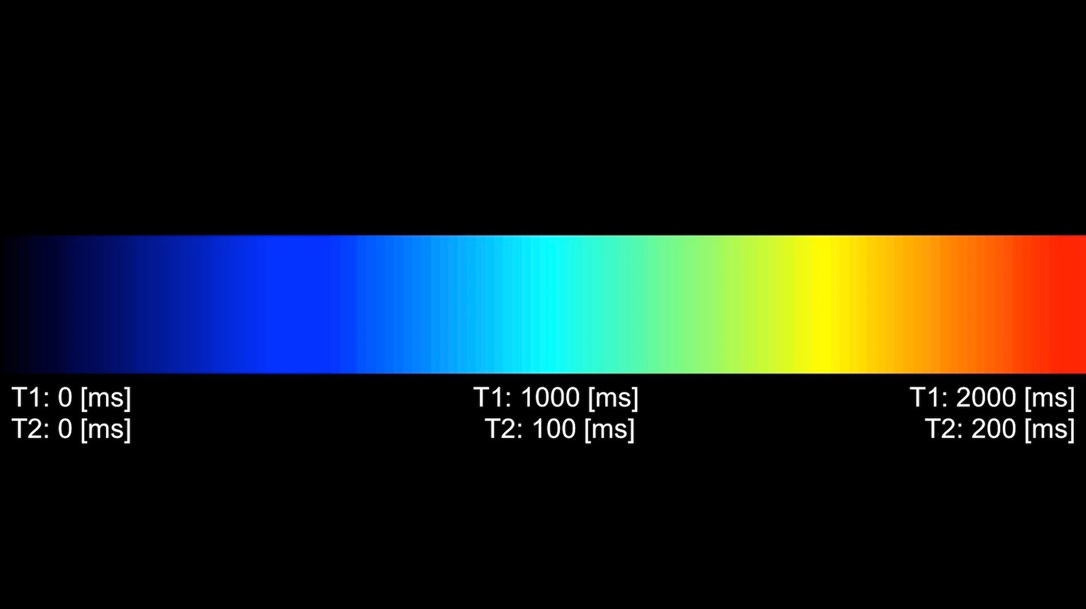


**Supplementary Figure 1:** Color-coding according to the T1- and T2-relaxation constants.

**
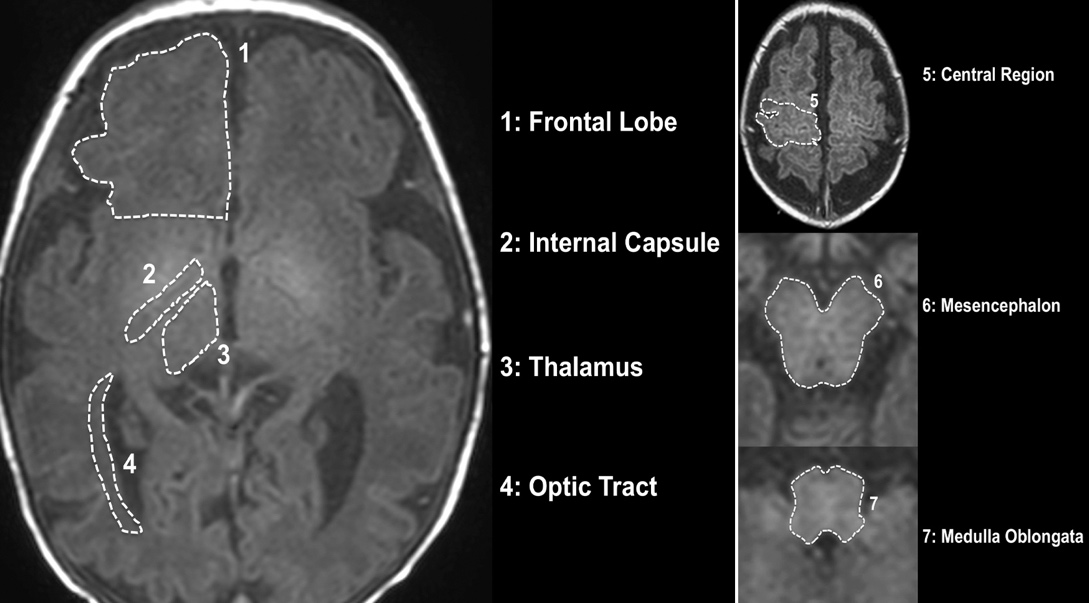
**

**Supplementary Figure 2:** Placement of regions of interest.


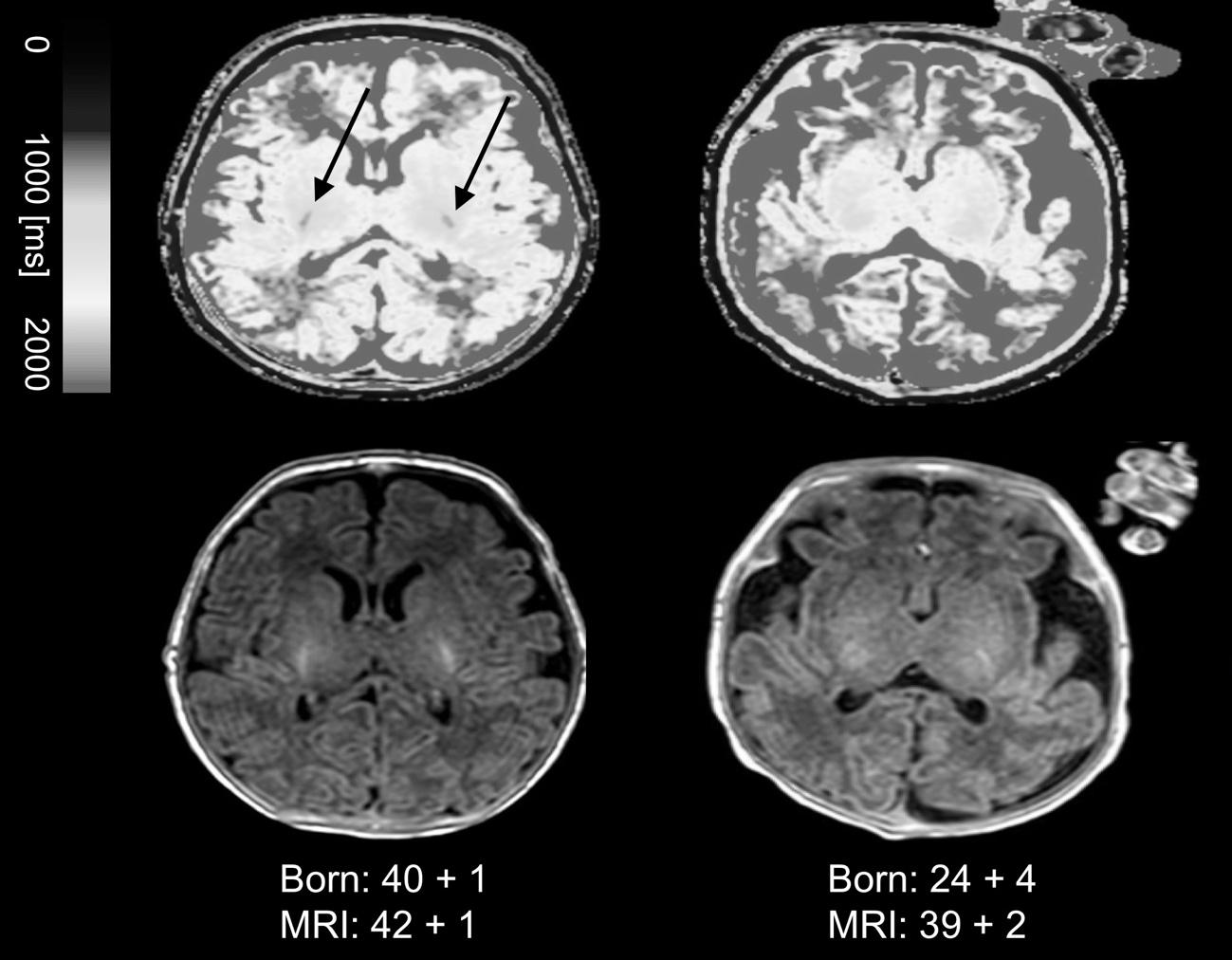


**Supplementary Figure 3:** Figure 1 Black/White.

**
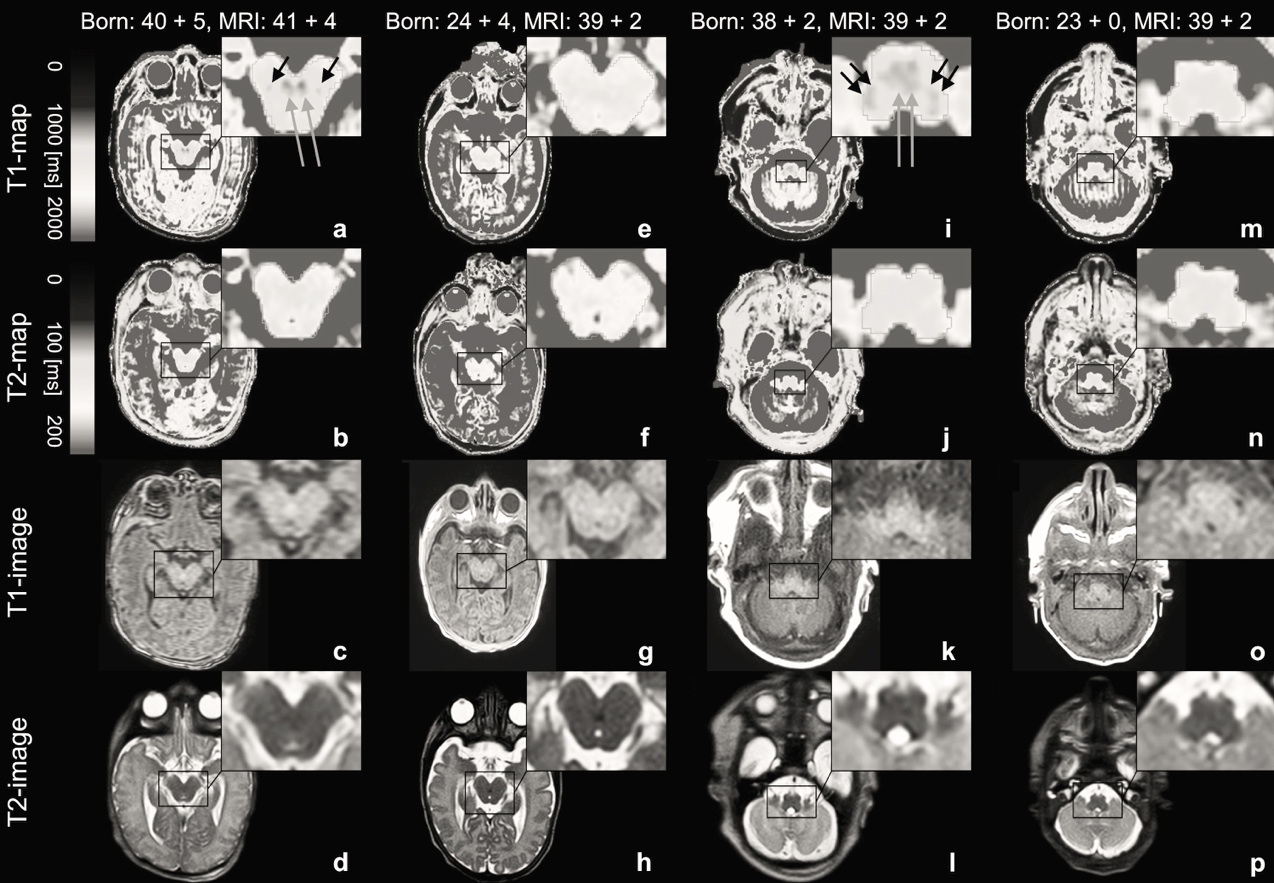
**

**Supplementary Figure 4:** Figure 2 Black/White.
